# Versatile members of the DNAJ family show Hsp70 dependent anti-aggregation activity on RING1 mutant parkin C289G

**DOI:** 10.1038/srep34830

**Published:** 2016-10-07

**Authors:** Vaishali Kakkar, E. F. Elsiena Kuiper, Abhinav Pandey, Ineke Braakman, Harm H. Kampinga

**Affiliations:** 1 University Medical Center Groningen, University of Groningen, Department of Cell Biology, Antonius Deusinglaan 1, 9713 AV, Groningen, The Netherlands; 2 European Research Institute for the Biology of Ageing, University of Groningen, University Medical Center Groningen, Antonius Deusinglaan 1, 9713 AV, Groningen, The Netherlands; 3 Utrecht University, Cellular Protein Chemistry, Bijvoet Center for Biomolecular Research, Padualaan 8, 3584 CH, Utrecht, The Netherlands

## Abstract

Parkinson’s disease is one of the most common neurodegenerative disorders and several mutations in different genes have been identified to contribute to the disease. A loss of function parkin RING1 domain mutant (C289G) is associated with autosomal-recessive juvenile-onset Parkinsonism (AR-JP) and displays altered solubility and sequesters into aggregates. Single overexpression of almost each individual member of the Hsp40 (DNAJ) family of chaperones efficiently reduces parkin C289G aggregation and requires interaction with and activity of endogenously expressed Hsp70 s. For DNAJB6 and DNAJB8, potent suppressors of aggregation of polyglutamine proteins for which they rely mainly on an S/T-rich region, it was found that the S/T-rich region was dispensable for suppression of parkin C289G aggregation. Our data implies that different disease-causing proteins pose different challenges to the protein homeostasis system and that DNAJB6 and DNAJB8 are highly versatile members of the DNAJ protein family with multiple partially non-overlapping modes of action with respect to handling disease-causing proteins, making them interesting potential therapeutic targets.

Parkinson’s disease (PD) is characterized by progressive accumulation of stable protein aggregates in the cytoplasm, named Lewy bodies, that lead to selective loss of dopaminergic neurons in the substantia nigra^1^,^2^. Several heritable forms of PD are related to mutations in the genes for α-synuclein (*SNCA*) and parkin (*PARK2*) that are associated with protein aggregation^1^,^3^,^4^. Parkin is an E3 ubiquitin ligase that plays a role in ubiquitination of several candidate substrate proteins and thereby targeting them for proteasomal degradation. Parkin is also involved in the elimination of damaged mitochondria through autophagy, called mitophagy^5^,^6^. Parkin contains an N-terminal ubiquitin-like (Ubl) domain, a central RING (Really Interesting New Gene) domain (RING0) and a C-terminal RING domain consisting of two RING finger motifs (RING1 and RING2) separated by an In-between-RING (IBR) domain^7^. A wide variety of *PARK2* mutations have been found, including exon deletions, duplications and triplications, missense, nonsense, and frameshift mutations^8^. Here, we focus on one of the first-reported mutations in the RING1 domain of parkin, the Cys^289^ to Gly (C289G) mutation, which is associated with an autosomal-recessive form of juvenile parkinsonism (AR-JP)^9^,^10^. Besides a loss of function, the C289G mutation results in alterations in parkin solubility and sequestration in aggresome-like protein aggregates and hence might also indicate a dominant, toxic gain-of-function phenotype^11^,^12^,^13^. Indeed, expression of parkin mutants in *Drosophila melanogaster* lead to neurodegeneration and motor impairments and carrying a single allele with the C289G mutant is associated with a higher risk of parkinsonism^14^,^15^,^16^. Formation of C289G parkin aggregates are likely due to loss of a conserved cysteine in the RING domain, which impairs interaction of the RING2 domain with RING0 and RING1 and hence affects the more compact arrangement in the protein. This leads to a disrupted protein structure, which renders it misfolded and inactive^17^.

Molecular chaperones play a crucial role in various ways in the prevention of aggregation of different mutant proteins. Heat shock proteins (HSPs) by virtue of their function as molecular chaperones act as the first line of defence against protein aggregation. Most HSPs recognize exposed hydrophobic regions of non-native proteins and hereby can prevent protein aggregation. In doing so, they not only assist in protein (re)folding but also in the degradation of misfolded client proteins by targeting them to the protein degradation machineries^18^,^19^. Given the capacity of molecular chaperones to prevent aggregation of misfolded proteins, and reminiscent of our previous work on polyglutamine (polyQ) aggregation^20^,^21^, they might be of use as therapeutic intervention to prevent aggregation of parkin C289G^20^,^22^,^23^,^24^,^25^,^26^,^27^,^28^,^29^. Two related members within the Hsp40 family of chaperones (the DNAJB subfamily), namely DNAJB6 and DNAJB8, were found to be excellent suppressors of protein aggregation in a polyQ disease cellular model, whilst several other members were less or not active^20^. DNAJB6 up-regulation in mouse brain delays polyQ aggregation, relieves symptoms, and prolongs lifespan^30^. Detailed analysis showed that both *in vitro* and in cells DNAJB6 was able to bind to polyQ containing polypeptides through an S/T rich stretch^30^ that competes with the hydrogen bonding necessary for formation of amyloid fibrils by β-hairpins^31^. In line, DNAJB6 prevents both the nucleation of Aβ peptides into amyloids and the incorporation of Aβ into pre-existing amyloid fibers^32^. Using the same cell model used for the polyQ protein aggregation, we here performed a screen for DNAJ proteins that could handle parkin C289G aggregation. Unlike for polyQ aggregation, all cytoplasmic DNAJs were found to be almost equally effective in preventing parkin C289G aggregation and this activity required a functional J-domain, meaning their functionality was dependent on interaction with Hsp70 s. However, for the anti-aggregation activity of DNAJB6 and DNAJB8 on parkin C289G the S/T rich stretch was found to be dispensable, indicating that polyQ and parkin aggregation occur via distinct routes. Our data further show that DNAJ proteins keep parkin C289G in a soluble, degradation-competent form, thus increasing the total amount (but not rate) of parkin C289G being degraded.

## Results

### Most DNAJA and DNAJB family members efficiently reduce parkin C289G aggregation

**Figure 1:**
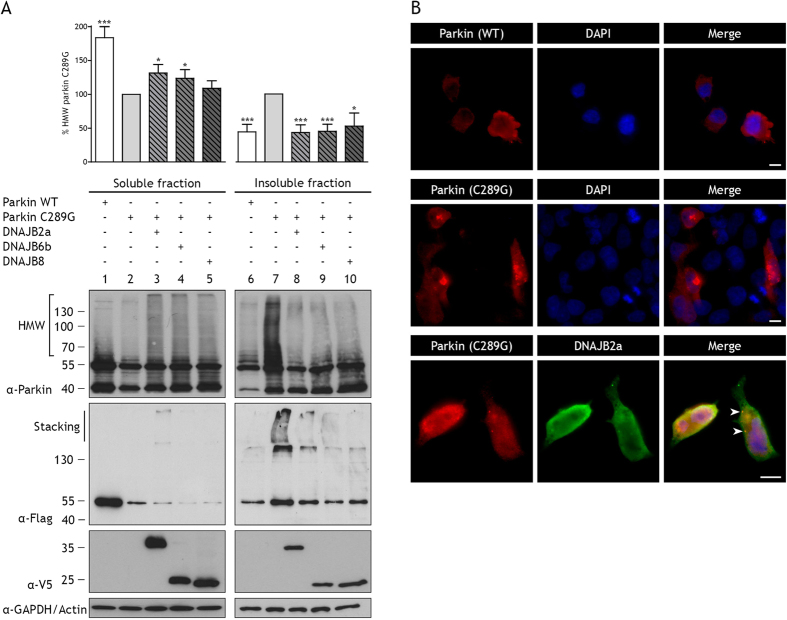
DNAJB2a, DNAJB6b, or DNAJB8 can prevent aggregation of parkin C289G. (**A**) HEK293 cells were transfected with flag-tagged parkin WT, flag-tagged parkin C289G, or co-transfected with flag-tagged parkin C289G and V5-tagged DNAJB2a, DNAJB6b, or DNAJB8. Expression of chaperones was induced with tetracycline. Triton X-100 (TX-100) soluble and insoluble fraction were obtained 24 hours after transfection. Parkin WT and C289G were assessed with anti-parkin and anti-flag antibodies on Western blot and analysed for parkin C289G high molecular weight species and normalised to parkin C289G (*p < 0.05; ***p < 0.001; n > 4 independent samples, mean ± SEM). In the TX-100 insoluble fraction, high molecular weight (HMW) species of parkin C289G are observed. Co-transfection with DNAJB2a, DNAJB6b, or DNAJB8 prevents formation of parkin C289G HMW species. Anti-parkin antibodies detect full-length parkin and an N-terminally truncated isoform (also removing the flag-tag), generated due to an internal start site, showing an extra band around 42 kDa^68^. The anti-flag antibodies recognize predominantly the full-length parkin protein. Expression of chaperones was detected with anti-V5 antibodies. GAPDH was used as a loading control for the soluble fraction. (**B**) Representative immunofluorescence pictures of cells co-transfected with flag-tagged parkin WT or parkin C289G (red) and V5-tagged chaperones (green). DAPI staining is shown in blue. Bar represents 10 μm. Parkin WT shows a diffuse pattern throughout the cytoplasm. Parkin C289G forms aggregates mainly concentrated into large perinuclear inclusions. Co-transfection with DNAJB2a reveals clearance of parkin C289G aggregates and co-localization of DNAJB2a with parkin C289G, indicated by arrowheads.

**Figure 2:**
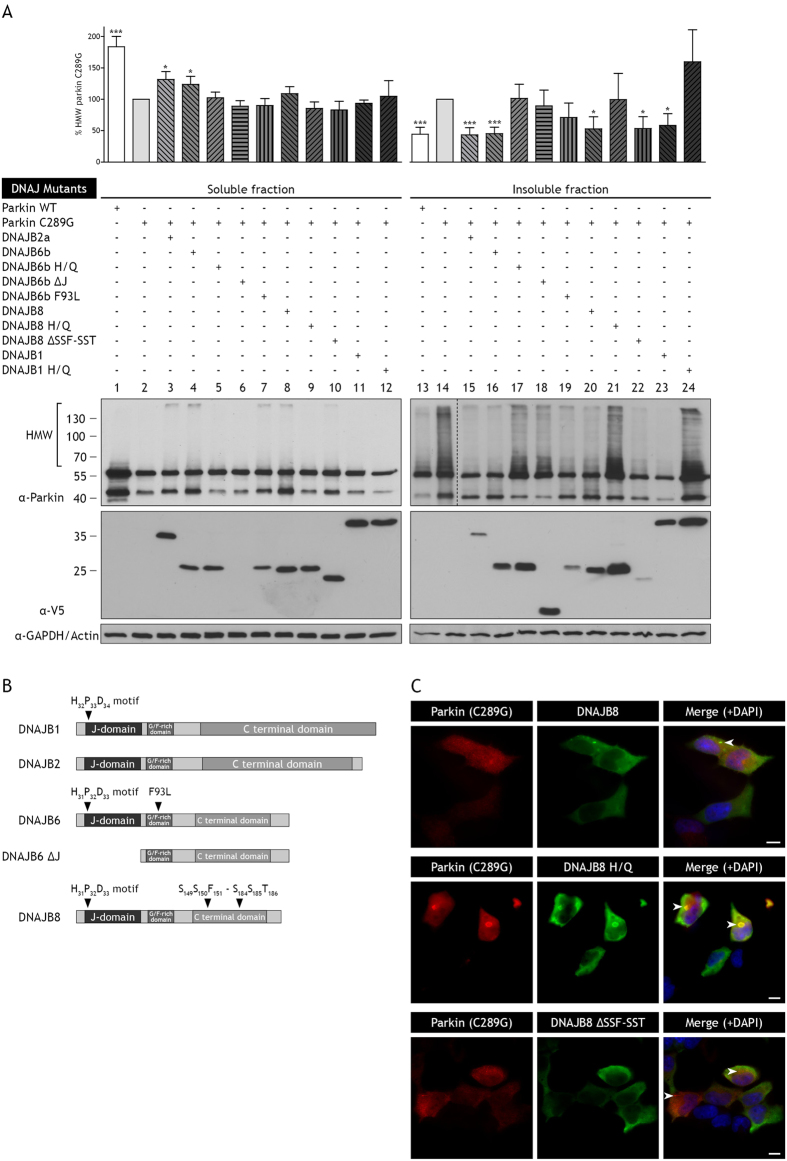
The anti-aggregation activity of DNAJ chaperones is dependent on the J-domain, not on the SSF-SST domain. (**A**) Aggregation of parkin C289G is prevented when co-transfected with DNAJB2a, DNAJB6b, DNAJB8, or DNAJB1. Mutations in the J-domain of the chaperones (H/Q) and deletion of the J-domain as a whole (ΔJ), results in the loss of prevention of aggregation of parkin C289G. DNAJB6b with a mutation in the G/F-rich region (F93L) retains its anti-aggregation activity. Deletion of the SSF-SST domain (ΔSSF-SST) of DNAJB8 does not affect its ability to prevent aggregation. Blots are analysed for parkin C289G high molecular weight species and normalised to parkin C289G (*p < 0.05; ***p < 0.001; n > 4 independent samples, mean ± SEM). (**B**) Schematic overview of DNAJB1, DNAJ2a, DNAJB6, and DNAJB8 and the mutants. (**C**) Representative immunofluorescence pictures of cells co-transfected with flag-tagged parkin C289G (red) and V5-tagged chaperones (green). DAPI staining is shown in blue. Bar represents 10 μm. Co-transfection with DNAJB8 shows a reduction in parkin C289G aggregates and co-localization of DNAJB8 with parkin C289G, indicated by arrowheads. Co-transfection with DNAJB8 H/Q still shows formation of parkin C289G aggregates mainly concentrated into large perinuclear inclusions, indicated by arrowheads. Parkin C289G and DNAJB8 H/Q co-localize but aggregate formation is not prevented. Co-transfection with DNAJB8 ΔSSF-SST reveals clearance of parkin C289G aggregates and in occasional small aggregations co-localization of DNAJB8 ΔSSF-SST with parkin, indicated by arrowheads.

Previously, we have shown that aggregation of polyQ proteins can be prevented most efficiently by DNAJB6b and DNAJB8, as well as DNAJB2a^20^, but less efficient by most other DNAJs. To test which DNAJ members are also able to reduce parkin C289G aggregation, HEK293 cells were transiently co-transfected with V5-tagged DNAJs and flag-tagged parkin wildtype (WT) or C289G mutant. Upon Triton X-100 (TX-100) lysis, most of the wild-type protein distributes to the TX-100 soluble fraction (Fig. 1A, lane 1 and 6), whereas parkin C289G ends up in the TX-100 insoluble fraction (Fig. 1A, lane 2 and 7). In addition, for parkin C289G a high molecular weight (HMW) smear in the TX-100 insoluble fraction of the cell lysate can be seen (Fig. 1A, lane 7), consistent with earlier work^6^ and indicative of parkin C289G aggregation. In parallel, the steady state levels of parkin C289G were lower than that of parkin WT (Supplementary Fig. S1). Furthermore, cells expressing parkin WT show uniform distribution of the protein whilst parkin C289G shows multiple large inclusions scattered throughout the cytoplasm (Fig. 1B, panel 1 and 2). Co-expression of DNAJB6b and DNAJB8 as well as DNAJB2a, decreased parkin C289G aggregation to a similar extent (Fig. 1A, lane 8, 9 and, 10), which was always paralleled by a reduction in the steady state levels of parkin C289G (Supplementary Fig. S1, see also below). Inclusion formation was also equally effectively reduced by DNAJB2a (Fig. 1B, panel 3) and DNAJB8 (Fig. 2C, panel 1). For DNAJB2a, these data are in line with the findings of Rose *et al*. using different cell lines^6^. Next, other members of the DNAJA and DNAJB family were tested for their ability to prevent aggregation of parkin C289G. Co-expression of most DNAJs were found to reduce insolubilization of parkin C289G (Supplementary Fig. S2A,B). Exceptions are DNAJA3, which is localized to mitochondria^33^,^34^, and DNAJB9, which is normally localized to the ER^33^,^35^ and was hardly expressed here. The inability of these chaperones to prevent parkin C289G aggregation must be attributed to their different localization than parkin, which is predominantly cytosolic and forms perinuclear inclusions. The finding that all cytoplasmic DNAJAs and DNAJBs tested were found to be almost equally effective in preventing parkin C289G aggregation, differs strongly from what we found for prevention of polyQ aggregation suggesting that polyQ and parkin C289G requires different handling by the protein quality control (PQC) system.

### A functional J-domain is critical for activity of DNAJ proteins against parkin C289G aggregation

To investigate the role of the different domains of DNAJ proteins in preventing parkin C289G aggregation, different mutants of the DNAJB6b and DNAJB8 chaperones were co-transfected with parkin C289G (Fig. 2A). DNAJ chaperones contain a highly conserved ~70 amino-acid J-domain with a histidine-proline-aspartic acid (HPD) motif, which is necessary for the interaction between DNAJs and Hsp70 s^36^. Here, we used DNAJ mutants in which the histidine residue in the HPD motif was substituted by a glutamine (H/Q) leading to a J-domain that can no longer bind to Hsp70 s. We also used a mutant of DNAJB6b in which the entire J-domain was deleted (ΔJ) (Fig. 2B). Mutations in the J-domain or deletion of the J-domain render the DNAJs incapable of preventing aggregation of parkin C289G (Fig. 2A, lane 17, 18, 21, and 24). Using DNAJB8 as a representative of DNAJ family, we confirmed this with immunohistochemistry, showing that in cells co-transfected with the H/Q mutant of DNAJB8, parkin C289G still forms large inclusions. Interestingly, unlike wild type DNAJB8, the DNAJB8 H/Q mutant was found to co-localize with parkin C289G aggregates, suggesting that it may be trapped here due to its inability to interact with Hsp70 (Fig. 2C, panel 2). Together, these data imply that Hsp70 interaction is required for all DNAJs to prevent parkin C289G aggregation.

We used a deletion mutant of DNAJB8 that lacks the serine/threonine (S/T) rich domain (ΔSSF-SST) at the C-terminal (Fig. 2B). In contrast to the crucial role of this domain in the prevention of polyQ aggregation^20^,^30^, the ΔSSF-SST mutant fully retained its anti-aggregation activity on parkin C289G as revealed by fractionation (Fig. 2A, lane 22) and immunofluorescence (Fig. 2C, panel 3). Besides the J-domain and the S/T domain mutants, we tested a DNAJB6b mutant with a missense mutation in the G/F-rich region (F93L). This F93L mutant is implicated in limb-girdle muscular dystrophy and had been suggested to be slightly impaired in delaying polyQ mediated aggregation^37^. The F93L mutant was still largely effective in preventing parkin C289G aggregation (Fig. 2A, lane 19), implying that this disease-causing mutation has little impact on the functional DNAJB6-Hsp70 interaction that deals with parkin C289G aggregates. These combined data further illustrate different chaperone activity requirements for dealing with either polyQ or parkin C289G aggregation.

### Hsp70 s are required for full prevention of parkin C289G aggregation

**Figure 3:**
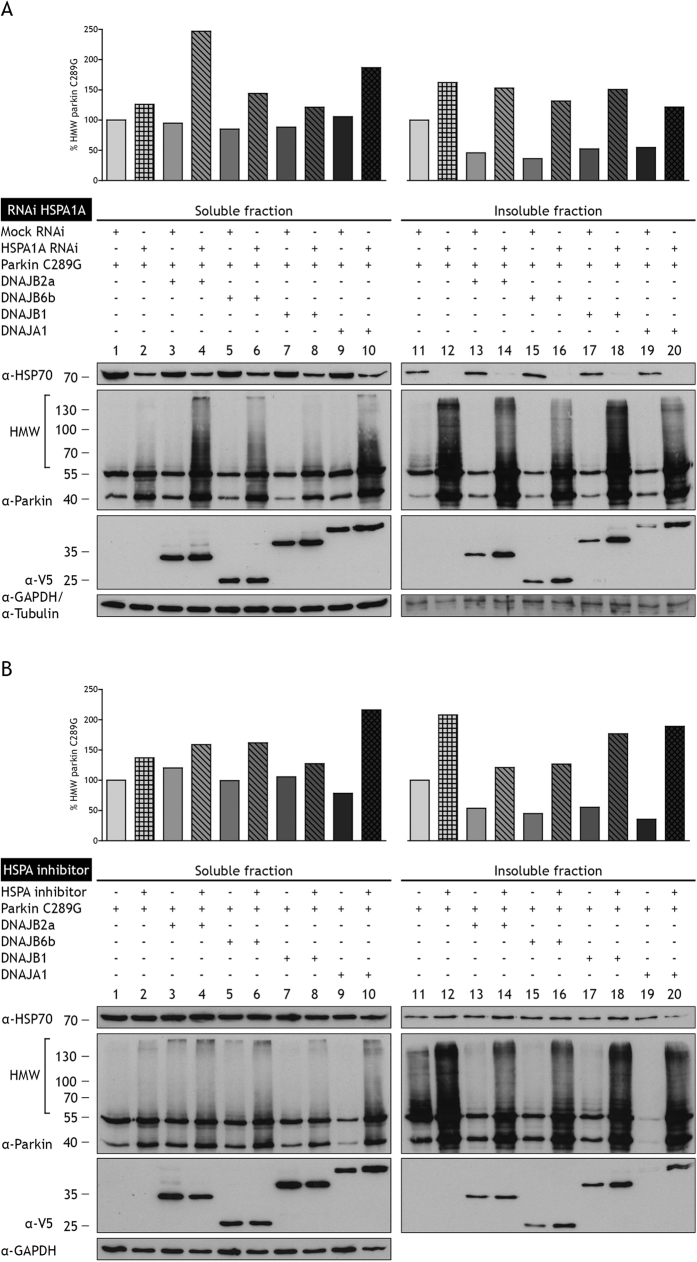
The anti-aggregation activity of DNAJ chaperones is dependent on Hsp70 s. (**A**) Knockdown of HSPA increases levels of TX-100 soluble and insoluble parkin C289G. In cells co-transfected with DNAJs, the HMW smear is still formed, indicating that DNAJs lose their ability to prevent parkin C289G aggregation. However, a reduction in the smear can be observed when parkin C289G is co-transfected with DNAJB2a, DNAJB6b, or DNAJA1 (not DNAJB1) compared to parkin C289G alone. There is an increase in the TX-100 soluble fraction when co-transfected with DNAJs, indicating more soluble parkin C289G under conditions of knockdown of HSPAs and suggesting some Hsp70-independent actions of the DNAJs prior to parkin insolubilization. (**B**) Hsp70 s are inhibited with VER-155008, an adenosine-derived HSPA inhibitor that targets the ATPase binding domain. Similar results are observed as in panel A.

To further consolidate that DNAJs prevent aggregation of parkin C289G via an interaction with Hsp70 s, we examined whether short interfering RNA (siRNA)-mediated knockdown of HSPA1A had an effect on the DNAJ mediated clearance of parkin C289G. Interestingly, the partial siRNA-mediated knockdown of HSPA1A alone already increases parkin C289G insolubilization considerably (Fig. 3A, lane 11 and 12), implying that constitutively expressed HSPA1A contributes to handling of parkin C289G to reduce its aggregation. In the siRNA-treated cells, the overexpressed DNAJs largely lost their ability to prevent parkin C289G aggregation. Yet, a small reduction in parkin C289G aggregation is still seen for DNAJB2a, DNAJB6b, or DNAJA1 (but not DNAJB1) (Fig. 3A, lane 14, 16, and 20 compared to lane 12). Under knockdown conditions more of the parkin C289G HMW smears were now detected in the TX-100 soluble fraction of DNAJB2a, DNAJB6b, and DNAJA1 co-expressing cells (Fig. 3A, lane 4, 6, 8, and 10), suggesting some Hsp70-independent actions of the DNAJs prior to parkin C289G insolubilization. When cells were treated with VER-155008, an adenosine-derived Hsp70 inhibitor that targets the ATPase binding domain of all Hsp70 s^38^, similar results were observed (Fig. 3B). These data suggest that DNAJs are capable in maintaining parkin C289G in a soluble state when Hsp70 s are depleted but that Hsp70 s are required for full prevention of parkin C289G aggregation.

From the above results so far, parkin C289G appears to be a substrate for the Hsp70 machine, in which upregulation of different DNAJs can enhance the cellular capacity for its degradation by keeping it more soluble, as long as sufficient Hsp70 activity is available (Figs 1, 2 and 3). Yet, upregulation of different members of the Hsp70 family alone did not result in reduced parkin C289G aggregation (Supplementary Fig. S3A, lanes 12–18)), suggesting that other factors are actually rate limiting. A minimal Hsp70 machine contains, besides DNAJs and HSPAs, also nucleotide exchange factors (NEFs)^33^. NEFs are a heterologous group of co-factors required for the dissociation of the substrate from HSPA and may also - in part - determine the fate of the substrate^33^, like HSPH2, which is able to remodel the Hsp70 machine to efficiently disaggregate aggregated proteins, and BAG3^39^, which has been implicated in dealing with aggregation of polyQ proteins. However, neither overexpression of the cytosolic NEFs (HSPH1, HSPH2, or HSPH3) (Supplementary Fig. S3B) nor the overexpression of the BAG family members (BAG1, BAG3, and BAG4) (Supplementary Fig. S3C) could reduce the aggregation of parkin C289G. So, within the background of endogenous expression levels of HSPs in HEK293 cells, overexpression of DNAJs seems the most effective way (i.e. rate limiting) to increase the efficiency of the Hsp70 machine to prevent aggregation of parkin C289G.

### DNAJs keep parkin C289G in a soluble, degradation-competent form

**Figure 4:**
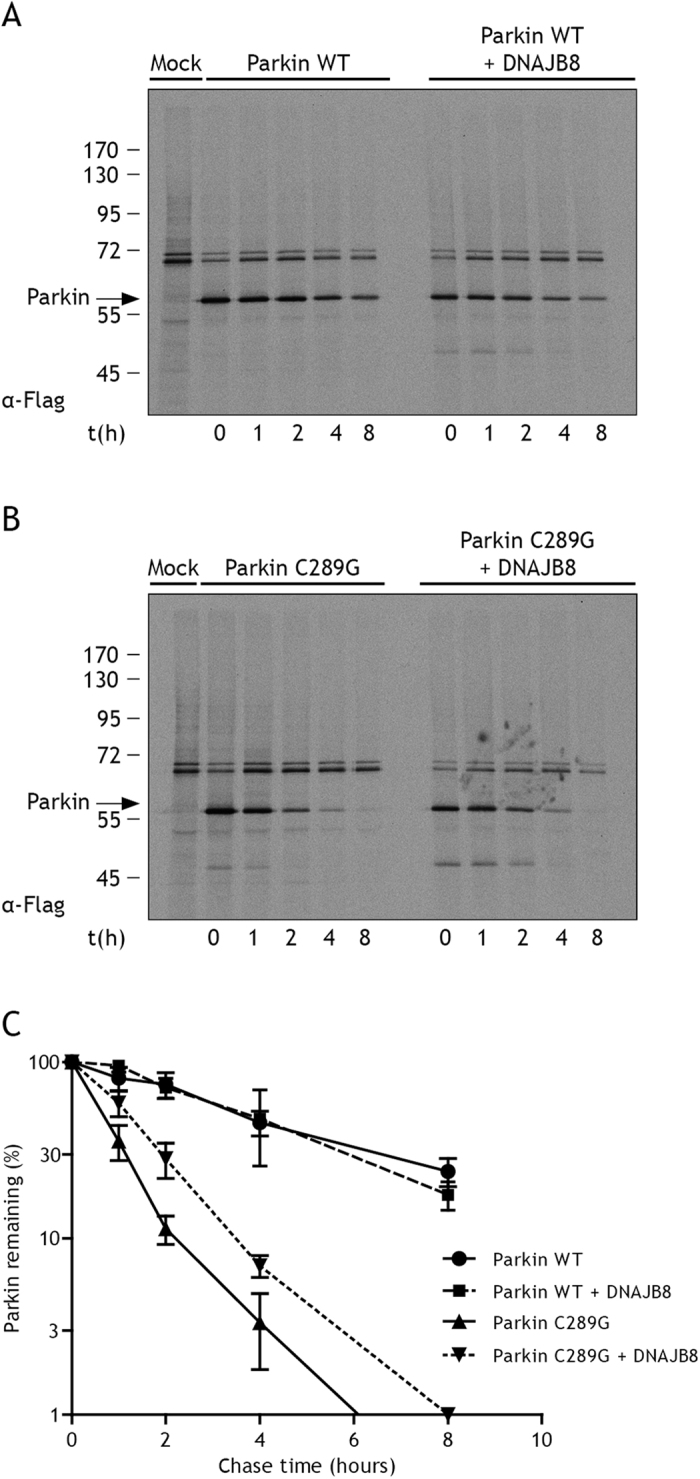
DNAJB8 keeps parkin C289G more in a soluble, degradation competent form. (**A**) Reduced autoradiograms of pulse chase labelling of flag-parkin. HeLa cells transfected with parkin WT, parkin C289G with or without DNAJB8 were pulse labeled with [35S]-labeled cysteine and methionine for 10 minutes and chased for the indicated time periods. Following detergent lysis, parkin WT and parkin C289G were immunoprecipitated with monoclonal flag antibody. Reduced samples were resolved by 10% SDS-PAGE. (**B**) Detergent soluble parkin WT or parkin C289G was quantified by phosphoimaging and data was plotted as a semi-log graph(n = 3 independent samples, mean ± SD).

To further examine how DNAJs affect parkin C289G aggregation, we performed pulse-chase experiments to monitor the fate of newly synthesized parkin. In the TX-100 soluble fraction, parkin C289G is more rapidly degraded than parkin WT (Fig. 4A,B), as shown before^11^ and indicative that it is recognized by the PQC system as misfolded. The expression of DNAJB8 (a representative of DNAJ family effective against parkin C289G aggregation) does not accelerate the rate of degradation of parkin WT or parkin C289G (Fig. 4C). However, DNAJB8 clearly reduced the fraction of parkin C289G (but not WT) that becomes TX-100 insolubilized in a 2-hour chase (Supplementary Fig. S4), meaning that in the presence of DNAJB8 more parkin C289G is kept competent for degradation over time, which is consistent with the reduced steady state levels of parkin C289G in cells co-expressing DNAJ proteins (Supplementary Fig. S1).

## Discussion

In the current study we show that, when upregulated in cells, all cytosolic members of the DNAJA and DNAJB subfamilies can suppress parkin C289G aggregation in an Hsp70 dependent manner. Hsp70 inhibition increased parkin C289G aggregation, even under conditions when DNAJs are overexpressed, but upregulation of HSPAs could not prevent an increase in parkin C289G aggregation. Further, overexpression of the cytosolic NEFs (HSPH1, HSPH2, HSPH3, BAG1, BAG3, and BAG4) did not lead to reduced parkin C289G aggregation. Comparing our current observations regarding the role of various chaperones on parkin C289G aggregation to our previous studies^20^,^21^,^39^ on aggregation of polyQ containing proteins, performed in the same cell model, reveals a number of intriguing features. First, the DNAJB6b and DNAJB8 chaperones that we previously identified as the most potent, but largely Hsp70-independent, suppressors of polyQ aggregation^20^, can also inhibit parkin C289G aggregation but do now fully depend on a functional Hsp70 machine. Second, the S/T-rich region in DNAJB6 and DNAJB8 that is crucial for preventing polyQ aggregation^20^,^30^, is dispensable for their protective action on parkin C289G. Third, DNAJB8 can keep parkin C289G in a degradation competent form, hereby reducing insolubilized material and increasing clearance of the mutant protein. This implies that different aggregation-prone, disease-causing proteins pose different challenges to the PQC systems and require specific chaperones for their handling and hence also highlight certain degree of functional differentiation between various families of HSP proteins.

The substantial differences between the chaperone requirements for two different aggregation-prone, disease-causing proteins can be explained by the fact that the aggregates caused by the two mutant proteins have different biochemical properties as revealed by detergent solubility. Whilst parkin C289G aggregates are TX-100 insoluble but largely SDS soluble (ref. 18, this report), polyQ aggregates are both TX-100 and SDS insoluble^20^,^40^,^41^,^42^,^43^. Further, the morphology of the aggregates is more dispersed for parkin C289G (ref. 18, this report) whilst polyQ proteins form more amyloid like inclusions^20^,^40^. Finally, whereas the Cys^289^ to Gly mutation in the RING1 domain of parkin leads to a disruption of the zinc coordination of the protein and thus to protein misfolding^44^, polyQ expansions within a full length huntingtin or ataxin-3 are not exposed and the full length proteins are not misfolded to an extent that they render the protein a target for accelerated degradation^45^,^46^. Rather, the full length polyQ expansion does not initiate aggregation until it is fragmented by the action of proteases^46^,^47^,^48^,^49^ or has undergone conformational changes such that flanking sequences open up or align the polyQ stretch for β-hairpin-mediated nucleation^31^,^50^,^51^. For the latter, we recently showed the S/T-rich region in DNAJB6 to be the crucial binding motif^30^, which is dispensable for its action on parkin C289G. Similar to DNAJ family members, differences were observed within the HSPB family of small HSPs: HSPB7 was the most effective in preventing polyQ aggregation and HSPB1 was ineffective^21^, while HSPB1 was very effective in preventing parkin C289G aggregation^52^. Finally, the mode of action of DNAJB6 and DNAJB8, which effectively reduced both types of aggregates, was different for polyQ proteins (largely Hsp70 independent) and parkin C289G (largely Hsp70 dependent). So, although several neurodegenerative diseases share protein aggregation as a common feature, the characteristics of the different aggregation-prone proteins impose a substantially different challenge on the cellular PQC system^29^,^53^.

We have previously shown that Hsp70 members are rather ineffective in preventing polyQ aggregation in HEK293 cells^20^. In contrast, in the same cells we now find that the activity and level of Hsp70 does matter for parkin C289G aggregation. Knocking down HSPA1A or pharmacological inhibition of “all” HSP70 s substantially aggravated parkin C289G aggregation (Fig. 3A,B). Yet, overexpression of the HSPA members or enhancing the ATPase activity of “all” Hsp70 s using the drug SW02^54^,^55^ did not lead to a reduction in parkin C289G aggregation (Supplementary Fig. S3A; Fig. S5), unlike what was found for other substrates, like the Alzheimer related proteins tau and amyloid-β^54^,^54^. These data show the differential effects of Hsp70 levels itself; they are not rate limiting in case of polyQ, are required but constitutively sufficiently expressed in case of parkin C289G, or required and rate limiting in case of tau. Moreover, overexpression of some HSP70 s (HSPA1L and HSPA9) even enhanced parkin C289G aggregation (Supplementary Fig. S3A). The latter is in line with the findings of the Hasson paper where knockdown of HSPA1L was found be associated with reduced parkin aggregation^56^. Together, this not only supports our conclusions that different aggregation-prone proteins require different chaperone handling^29^, but also suggests that besides DNAJs and NEFs, also the diverse members of the Hsp70 family can determine the functional specificity of Hsp70 machines^33^. Although this may seem striking giving the large sequence identity, the similar peptide recognition motifs, and biochemical activity that these Hsp70 members are assumed to have^57^,^58^, work by the Frydman group already suggested that distinct Hsp70 members may act in different chaperone networks, one predominantly dealing with protein translation and one with (environmental) stress^59^. Further support for functional differentiation between the Hsp70 family members was provided by the group of Jäättelä^60^ and our own findings that e.g. HSPA6 was largely ineffective in refolding unfolded substrates^61^.

**Figure 5:**
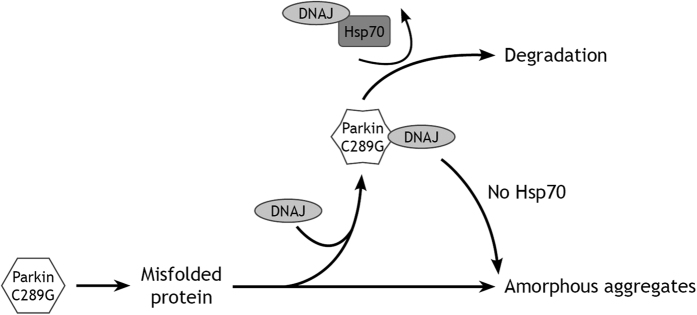
Model for the interaction of DNAJs with HSPAs for the handling of parkin C289G. DNAJs can primarily and transiently bind with the misfolded and ubiquitinated polypeptides of parkin C289G at an early stage (holding activity). In this way parkin C289G can be kept soluble and can be delivered via the Hsp70 s (HSPA1A and/or HSPA14) for degradation. When DNAJs are not sufficiently present or when Hsp70 s are depleted or inhibited, protein degradation fails and parkin C289G forms aggregates.

What is the mechanism through which all the DNAJs can suppress parkin C289G aggregation? For DNAJB2a, it has been suggested that prevention of parkin C289G aggregation was associated with restoring its function in mitophagy^6^, implying that such anti-aggregation effects are associated with a DNAJ/HSP70 assisted folding of the misfolded mutant. We indeed confirmed that parkin C289G shows a loss of function phenotype, as it no longer associated with mitochondria upon cellular treatment with the mitochondrial uncoupler CCCP (Supplementary Fig. S6A, panel 3 and 4; Fig. S6B). However, co-expression of DNAJBs did not restore this loss of function phenotype (Supplementary Fig. S6A, panel 5–7). The apparent difference between our data and those of the Cheetham group^6^ might be explained by the fact that the cells we used in our study (HeLa) express near to no endogenous wild type parkin, whilst the SK-N-SH cells (used by the Cheetham group) do^62^. This could imply that the aggregating mutant has dominant negative effects on the wild-type protein and that preventing such aggregation alleviates this effect to some extent. So, rather than refolding the mutant, our data suggest that DNAJs can maintain parkin C289G in a degradation-competent state. Whilst this does not accelerate the *rate* of mutant parkin degradation (Fig. 4A), the amount of parkin C289G that is degraded is increased in the presence of additional DNAJ protein expression (Fig. 4B), resulting in lower levels of parkin C289G (Fig. 1; Supplementary Fig. S1). This effect was not seen for parkin WT levels (Supplementary Fig. S1), indicating that this is related to the specific recognition of the misfolded species. The effect on parkin C289G was also lost when co-expressing the various DNAJ H/Q mutants that cannot interact with Hsp70 (Fig. 2), implying that Hsp70 is required for this action. Furthermore, as revealed by our western analysis, parkin C289G is present as a high molecular weight smear in the TX-100-insoluble fraction, consistent with other studies that the mutant is polyubiquitinated^11^,^63^, but has aggregated before being degraded. It is interesting to note that DNAJs were also still slightly effective under conditions of HSPA1A knockdown and that this was paralleled by an increase of high molecular weight species of parkin C289G in the TX-100 soluble fraction (Fig. 3A,B). Based on this, we propose a model for the handling of parkin C289G by the Hsp70 machinery (Fig. 5) in which the DNAJs primarily and transiently bind misfolding and aggregating polypeptides of parkin C289G, keeping them soluble for degradation (holding activity) via transfer by Hsp70 s. The holding activity of DNAJB1 may be the weakest of the ones tested, explaining why it lacks any effect when it when Hsp70 is depleted or inhibited.

Overall, our data further strengthens the concept that DNAJ proteins may be an attractive target in prevention of aggregation of disease-causing proteins. Furthermore, DNAJ upregulation does not seem to affect the general chaperone network (data not shown), which may have adverse effects^64^, and may be targeted for strong and specific interventions in various protein aggregation diseases. It was even more striking that DNAJB6, which we already identified as suppressor of amyloid-beta *in vitro*^32^, polyQ aggregation *in vitro*^65^, in cells^20^, and mice *in vivo*^30^ was also effective in suppressing parkin C289G aggregation in cells (this report), albeit with differential domain structure requirement. This suggests that DNAJB6 is a central and versatile player in PQC and thus attractive target in several neurodegenerative diseases including in Parkinson’s disease.

## Materials and Methods

### Cell line, cell culture, and transient transfections

HEK293 stably expressing the tetracycline (tet) repressor (Flp-In T-REx HEK293, Invitrogen) were grown in DMEM (GIBCO) plus 10% FCS (Greiner Bio-one), 100 units/ml penicillin, and 100 mg/ml streptomycin (Invitrogen), and for Flp-In TREx HEK293 cells, 5 mg/ml Blasticidine (Invitrogen). For transient transfections, cells were grown to 70–80% confluence in 35 mm diameter dishes coated with 0.001% poly-L-lysine (Sigma) and/or on coated coverslips for confocal microscopy analyses. Cells were transfected with Lipofectamine (Invitrogen) according to the manufacturer’s instructions with a 1:1 ration of parkin and molecular chaperones. When transfecting with molecular chaperones tetracyclin was added to the medium. For transfection with siRNA, Lipofectamine2000 (Invitrogen) was used according to the manufacturer’s protocol before transfection with Lipofectamine. For inhibition of Hsp70 activity, VER-155008 (Sigma) at a 40 uM concentration was added to cells immediately after transfection and then cells were further incubated for 8 hours.

### Gene cloning and generation of mutants

Information about the (construction of) the chaperone plasmids used in this study is described in Hageman *et al*.^20^. Information on the plasmid used for parkin is described in Ardley *et al*.^66^.

### Protein extraction to obtain soluble and insoluble fractions for Western blotting

24 hours after transfection cells were washed once in cold phosphate-buffered saline (PBS) and lysed in 200 μL 1% Triton X-100 in PBS, containing 1% complete protease inhibitor cocktail for mammalian cells (PIC) (Roche Applied Science) for 15 minutes on ice. Cell lysates were scraped and centrifuged at 14000 rpm (Eppendorf, rotor F45-30-11) for 15 minutes at 4 °C. Supernatants were transferred to eppendorfs (Triton X-100 soluble fraction). Pellets were resuspended in 200 μL 1% SDS in PBS, containing 1% PIC and sonicated before centrifugation at 14000 rpm (Eppendorf, rotor F45-30-11) for 15 minutes at 4 °C. Supernatants were collected (Triton X-100 insoluble fraction). Samples were diluted in 2x SDS-sample loading buffer with 20% β-mercaptoethanol (Sigma) to a final concentration of 1x and were left unboiled. Samples were used immediately or kept frozen at −20 °C.

### Western blot analysis

From the unboiled samples, 10 μl of soluble fraction, 10 μl of pellet fraction and 3 μl PageRuler Prestained Protein Ladder (Thermo Scientific) were loaded on 12.5% SDS-PAGE gels. SDS-PAGE was performed using the BioRad Mini-PROTEAN 3 system. After SDS-PAGE, proteins were transferred to nitrocellulose membranes by wet electrotransfer with the BioRad Mini-PROTEAN 3 system. The transfer was carried out for 1.5 hour at 100 V. To prevent non-specific protein binding, membranes were incubated in 5% (w/v) non-fat dried milk in PBS-Tween (PBS-T) for one hour at room temperature. Membranes were washed three times 5 minutes in PBS-T and incubated for at least one hour with mouse anti-parkin antibody (Cell Signaling Technology) at a 1:1000 dilution, mouse anti-flag antibody (Sigma-Aldrich) at a 1:2000 dilution, mouse anti-V5 antibody (Invitrogen) at a 1:5000 dilution, mouse anti-Hsp70 (Enzo Life Sciences) at a 1:5000 dilution, and mouse anti-GAPDH (Fitzgerald) at 1:10000 dilution. Membranes were washed three times 10 minutes and incubated for one hour in anti-mouse HRP-conjugated secondary antibody (GE Healthcare) at an 1:5000 dilution in PBS-T. After incubation, membranes were washed three times 10 minutes in PBS-T and enhanced chemiluminscence detection was performed using the ECL Western Blotting substrate kit (Thermo Scientific) using 750 μL solution 1, mixed with 750 μL solution 2 per membrane. Quantifications were performed using Gelpro software and graphs were generated using GraphPad prism. Statistical significance was analysed using an independent Student’s t-test, p < 0.05 was considered statistically significant. Values are expressed as the mean ± SEM.

### Immunolabeling and microscopy

24 hours after transfection, indirect immunofluorescence of the flag-tag and the V5-tag was performed to detect the exogenously expressed parkin and chaperones. Cells were fixed with 3.7% formaldehyde for 15 minutes, washed three times 5 minutes with PBS, permeabilized with 0.2% Triton-X100 and blocked 10 minutes with 0.1% glycine and 30 minutes with 0.5% BSA in PBS. Incubation with rabbit anti-V5 antibody (Sigma-Aldrich) and mouse anti-flag antibody (Sigma- Aldrich) at a 1:200 dilution in PBS-T was performed overnight at 4 °C. Cells were washed three times 5 minutes with PBS-T followed by a 1 hour incubation with Alexa Fluor 488 anti-rabbit (Invitrogen) and Alexa 594 anti-mouse (Invitrogen) secondary antibodies at a 1:1500 dilution in PBS-T. To visualize nuclei, cells were stained 10 minutes with 0.2 μg/ml 4’,6-diamidino-2-phenylindole (DAPI) (Thermo Fisher Scientific). Cover slips were mounted in Citifluor (Agar Scientific). Images of flag, V5 and DAPI fluorescence were obtained using a Leica microscope (Leica DM6000 M) with a 63X oil lens. The captured images were processed using Leica Software and Adobe Photoshop CS6.

### Pulse-chase analysis and immunoprecipitation

HeLa cells, approximately 24 hours post-transfection, were used for the pulse-chase analysis, which was carried out as described before^67^. Briefly, cells were pulse labeled for 10 minutes with 55 μCi/35 mm dish of Express ^35^S protein labelling mix (Perkin Elmer). The chase was carried out with excess cold cysteine and methionine at indicated times. After the chase, cells were lysed in ice-cold lysis buffer (0.5% Triton X-100 in 20 mM MES, 30 mM Tris-Cl pH 7.5, 100 mM NaCl) containing 1 mM PMSF, and 10 μg/ml each of chymostatin, leupeptin, antipain and pepstatin. Following pelleting of nuclei, supernatant was transferred to Protein-G sepharose beads (GE Healthcare) that had been preincubated for one hour at 4 °C with monoclonal anti-flag antibody (Sigma). After overnight immunoprecipitation, immunoprecipitates were washed twice at room temperature with lysis buffer for 10 minutes. The immunoprecipitates were resuspended in 10 mM Tris-Cl and 1 mM EDTA, pH 6.8 before adding 2x concentrated sample buffer (final concentrations 200 mM Tris-Cl pH 6.8, 3% SDS, 10% glycerol, 1 mM EDTA, 0.004% bromophenol blue, 25 mM DTT). Samples were heated for 5 minutes at 95 °C and analyzed using 10% SDS-PAGE. Dried gels were exposed to Phosphorimager screens and analyzed by Typhoon FLA 7000 (GE Healthcare). Quantifications were performed using ImageQuant TL7.0 software (GE Healthcare) and the graph was generated using GraphPad prism. Figures were obtained by exposing the dried gels to Carestream Kodak MR BioMax films.

## Additional Information

**How to cite this article**: Kakkar, V. *et al*. Versatile members of the DNAJ family show Hsp70 dependent anti-aggregation activity on RING1 mutant parkin C289G. *Sci. Rep.* **6**, 34830; doi: 10.1038/srep34830 (2016).

## Supplementary Material

Supplementary Information
