# Supplementary material for: Versatile members of the DNAJ family show Hsp70 dependent anti-aggregation activity on RING1 mutant parkin C289G
Source: Sci Rep. 2016 Oct 7;6:34830. doi: 10.1038/srep34830 (PMC5054386; doi:10.1038/srep34830)
Supplement: Supplementary Information [file srep34830-s1.pdf]

Versatile members of the DNAJ family show Hsp70 dependent anti-aggregation activity on RING1 mutant parkin C289G

Vaishali Kakkar[1#], E.F. Elsiena Kuiper[1,2#], Abhinav Pandey[3], Ineke Braakman[3], Harm H. Kampinga[1*]

[#] These authors contributed equally to this work

[1] University Medical Center Groningen, University of Groningen, Department of Cell Biology, Antonius Deusinglaan 1, 9713 AV, Groningen, The Netherlands.

[2] European Research Institute for the Biology of Ageing, University of Groningen, University Medical Center Groningen, Antonius Deusinglaan 1, 9713 AV, Groningen, The Netherlands.

[3] Utrecht University, Cellular Protein Chemistry, Bijvoet Center for Biomolecular Research, Padualaan 8, 3584 CH, Utrecht, The Netherlands.

[*] Correspondence should be addressed to H.H.K. (email: h.h.kampinga@umcg.nl)

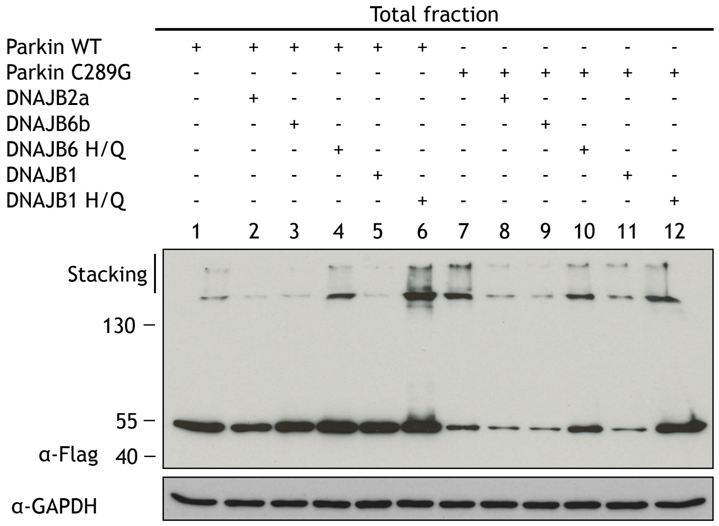

Supplementary Figure S1. Reduced total protein levels of parkin C289G compared to parkin WT and in cells co-expressing DNAJ proteins. HEK293 cells were transfected with flag-tagged parkin WT, flag-tagged parkin C289G, or co-transfected with V5-tagged DNAJB2a, DNAJB6b, or DNAJB1 and total TX-100 cell lysates were obtained 24 hours after transfection. Total levels of soluble parkin WT remain the same when DNAJs are overexpressed (lane 1-6). Total parkin C289G soluble levels are lower than parkin WT and are even further decreased upon overexpression of different DNAJs (lane 7-12).

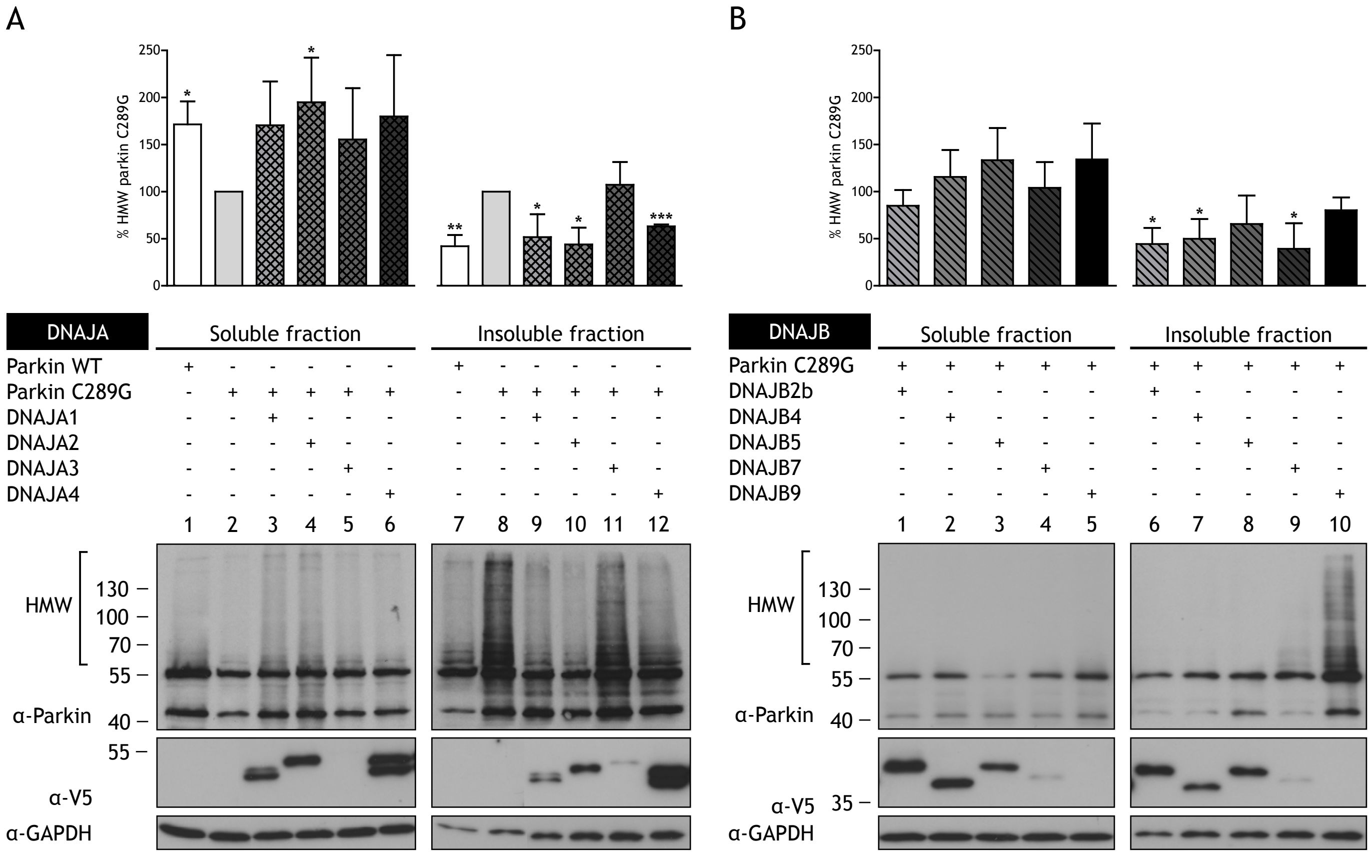

Supplementary Figure S2. Chaperones containing a J-domain can prevent aggregation of parkin C289G. (A) Accumulation of parkin C289G is prevented when co-transfected with members of the DNAJA subfamily (*=p<0.05; **=p<0.01; ***=p<0.001; n>3 independent samples, mean ± SEM). DNAJA3, which is localized to mitochondria is an exception. (B) Co-transfection with DNAJB chaperones could also prevent parkin C289G aggregation (*=p<0.05; **=p<0.01; ***=p<0.001; n>3 independent samples, mean ± SEM). DNAJB9 shows no prevention, due to localization of the chaperone to the ER. Expression of chaperones was detected with anti-V5 antibodies. GAPDH was used as a loading control for the soluble fraction.

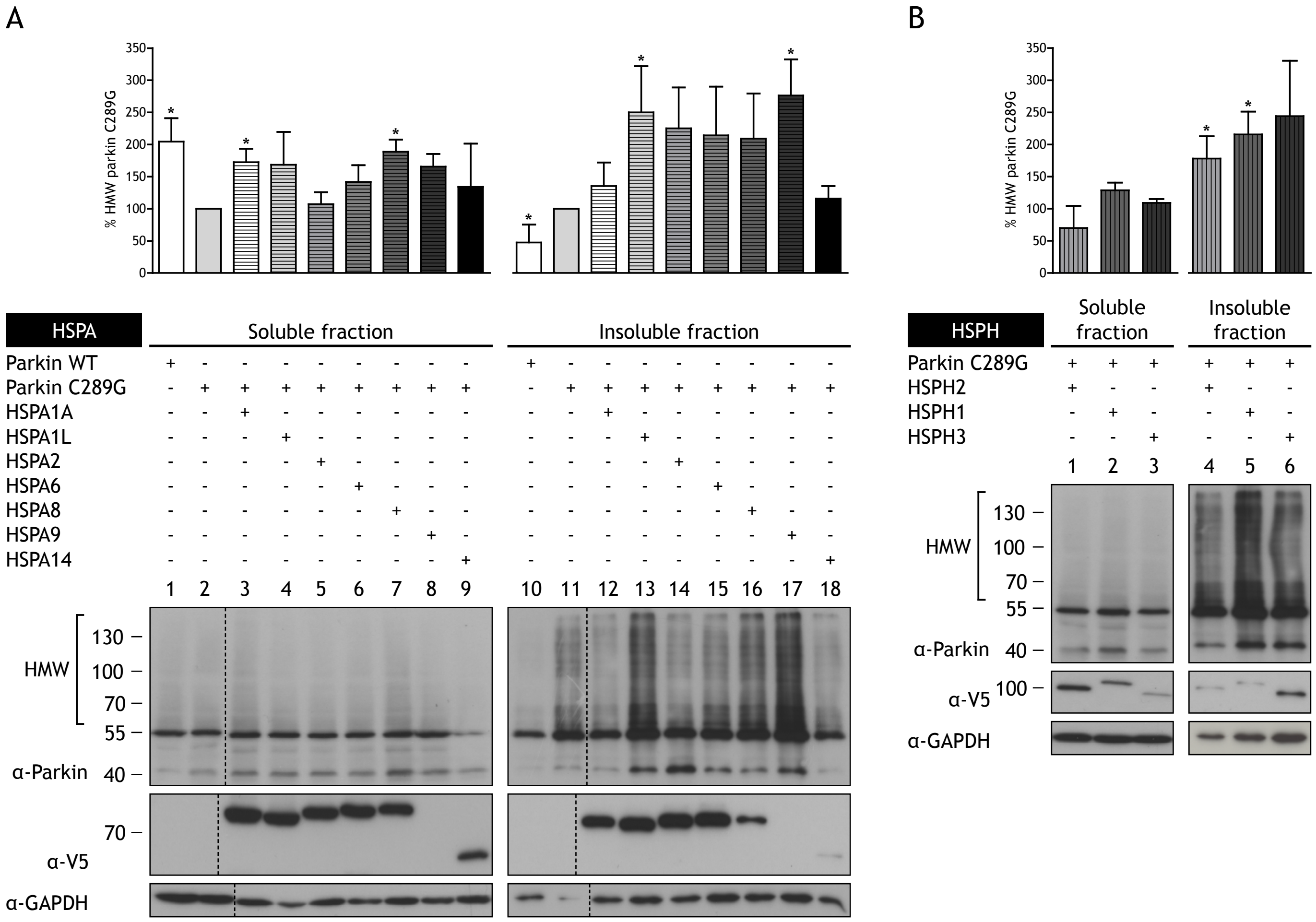
A
B
% HMW parkin C289G
350
300
250
200
150
100
50
0
HSPA
Soluble fraction
Insoluble fraction
Parkin WT
Parkin C289G
HSPA1A
HSPA1L
HSPA2
HSPA6
HSPA8
HSPA9
HSPA14
1 2 3 4 5 6 7 8 9
10 11 12 13 14 15 16 17 18
HMW
130
100
70
55
40
α-Parkin
α-V5
α-GAPDH
HSPH
Soluble fraction
Insoluble fraction
Parkin C289G
HSPH2
HSPH1
HSPH3
1 2 3 4 5 6

C

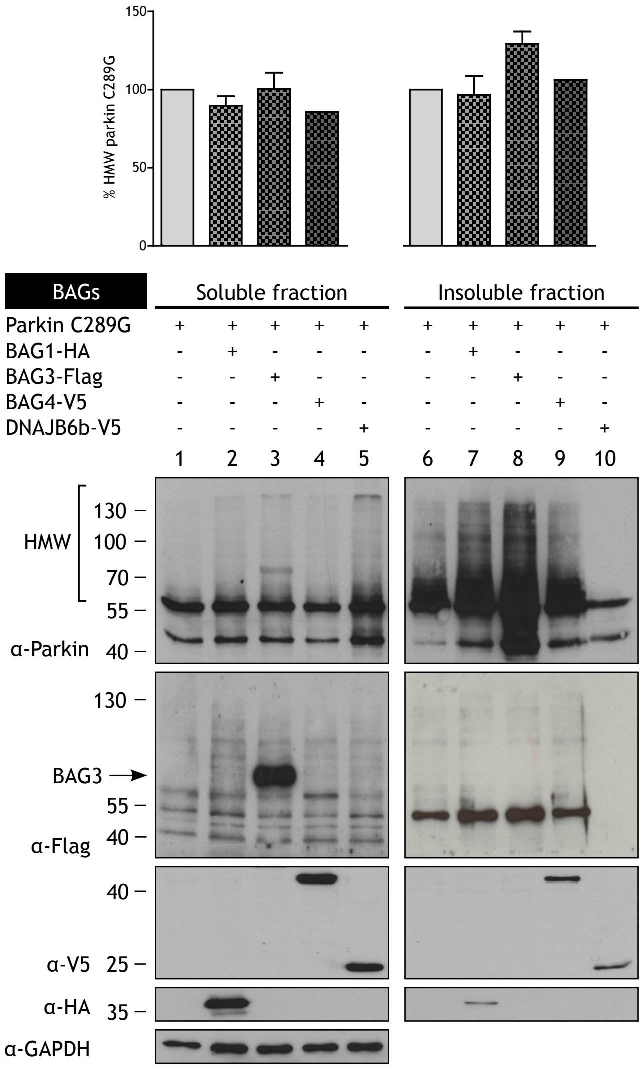

Supplementary Figure S3. Chaperones of the HSPA, HSPH, and BAG families are not capable of clearing mutant parkin aggregation. (A) Co-transfection with members of the HSPA family reveals that they cannot prevent aggregation of parkin C289G. Co-transfection of none of the HSPA members caused a decrease in the HMW species of parkin C289G. Blots are analysed for parkin C289G high molecular weight species and normalised to parkin C289G (*=p<0.05; **=p<0.01; ***=p<0.001; n=3 independent samples, mean ± SEM). (B) Co-transfection with members of the HSPH family shows no prevention of aggregation of parkin C289G (*=p<0.05; **=p<0.01; ***=p<0.001; n=3 independent samples, mean ± SEM). (C) Co-transfection with BAG1 or BAG3 shows no prevention of aggregation of parkin C289G (*=p<0.05; **=p<0.01; ***=p<0.001; n=2 independent samples, mean ± SEM).

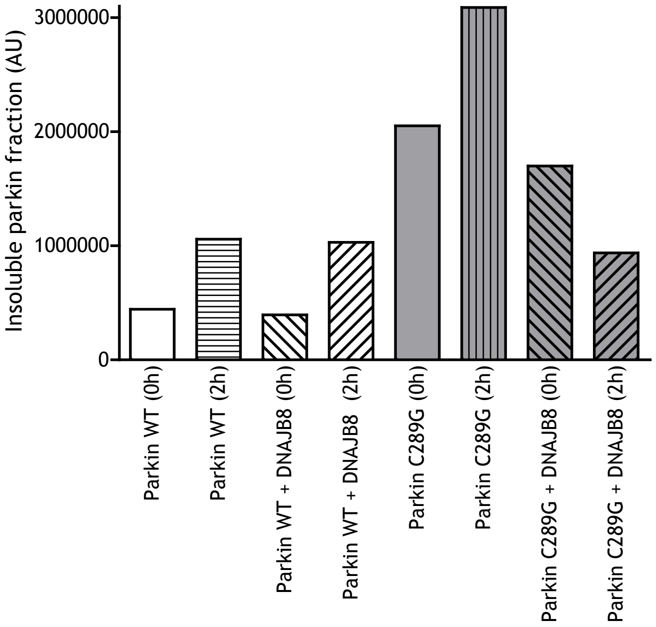

Supplementary Figure S4. DNAJB8 reduces the fraction of insoluble parkin C289G. HeLa cells transfected with parkin WT, parkin C289G with or without DNAJB8 and were pulse labeled with [35S]-labeled cysteine and methionine for 10 minutes and chased for two hours. Parkin WT and parkin C289G were immunoprecipitated from the insoluble pellet fractions with a monoclonal flag antibody. Reduced samples were resolved by 10% SDS-PAGE. Detergent insoluble parkin WT or parkin C289G was quantified by phosphor-imaging and data was plotted as insoluble fraction (in arbitrary units (AU)).

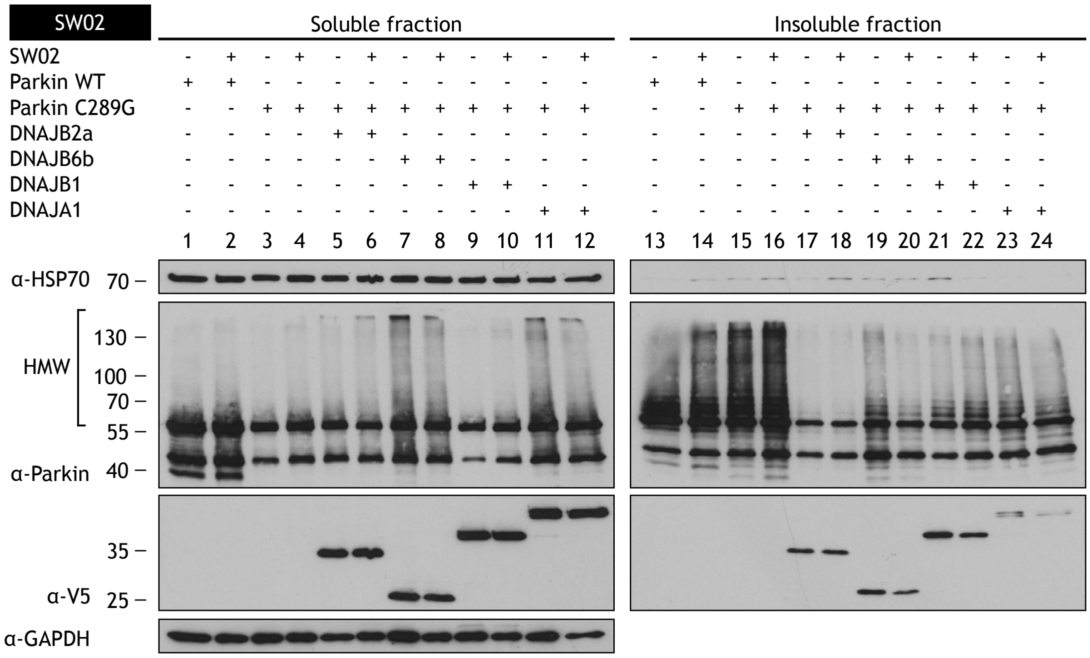

Supplementary Figure S5. Activation of the ATPase activity of endogenous Hsp70s with SW02 does not have an effect on parkin C289G aggregation. In HEK293 cells TX-100 soluble and insoluble levels of parkin C289G, alone and co-transfected with DNAJB2a, DNAJB6b, DNAJB1, or DNAJA1, remain the same when the ATPase activity of the Hsp70s is increased with the compound SW02.

A

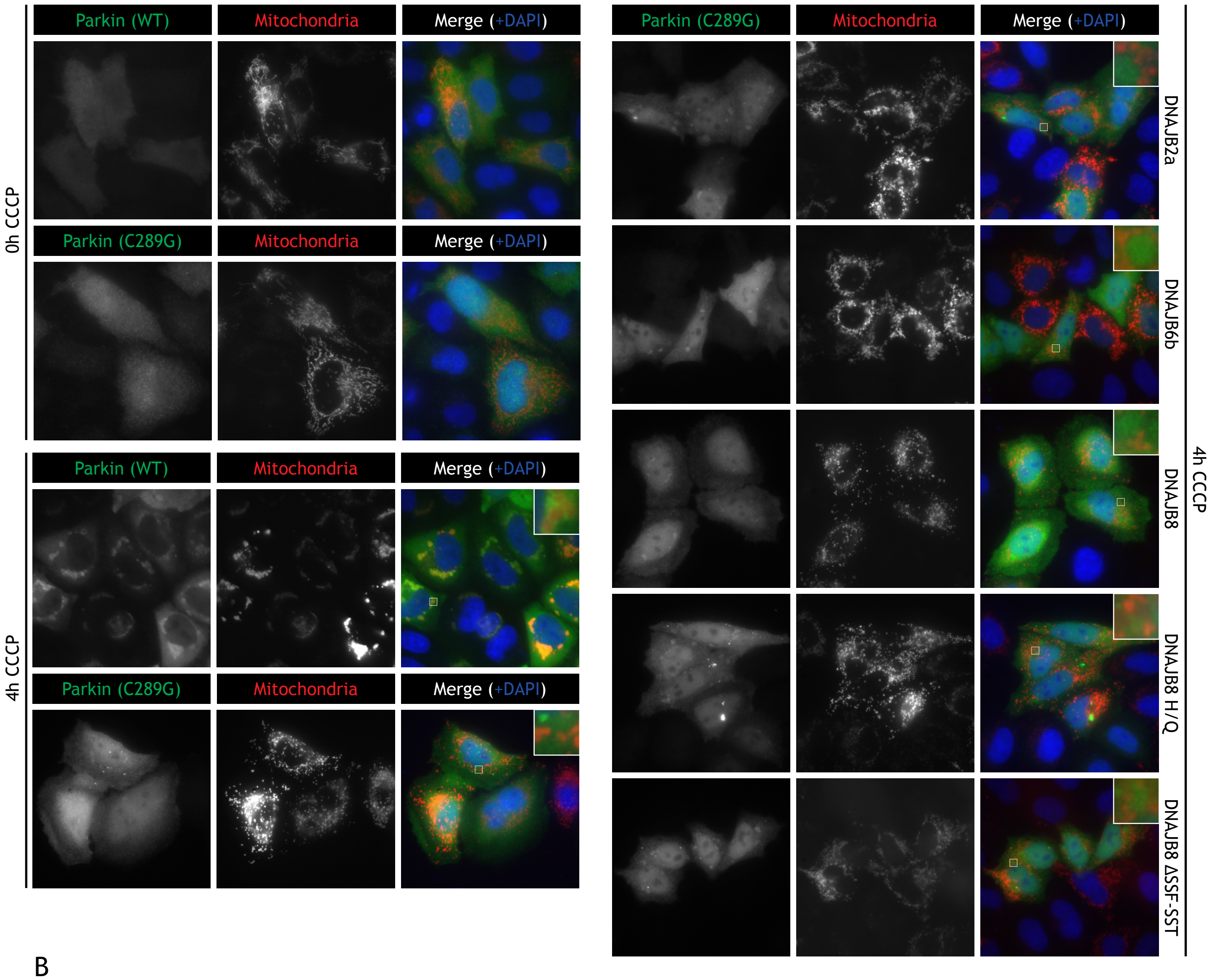

B

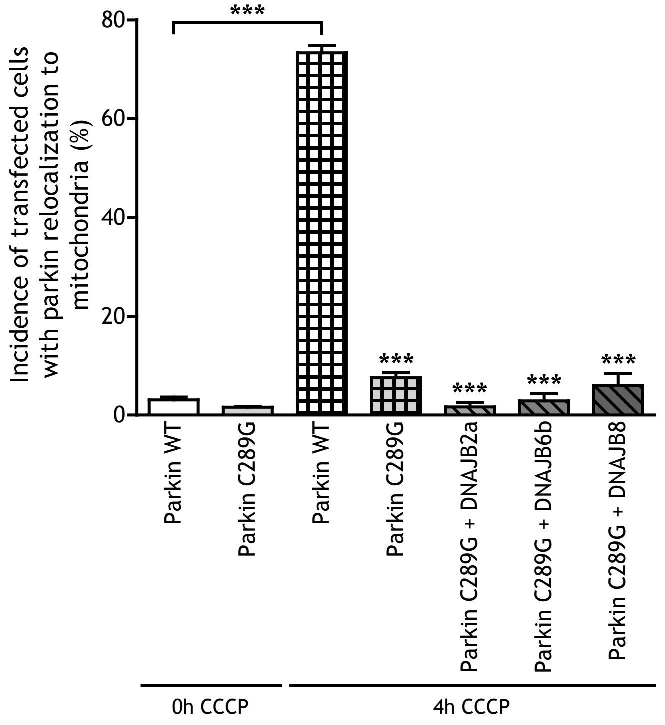

Supplementary Figure S6. Mutant Parkin C289G does not traffic to depolarized mitochondria and co-expression with DNAJBs does not correct this defect. (A) Representative immunofluorescence pictures of HeLa cells co-transfected with flag-tagged parkin WT or parkin C289G (green), mito-dsRed (red), and chaperones as indicated. DAPI staining is shown in blue. Upon treatment with the mitochondrial uncoupler CCCP, parkin WT relocalizes to the mitochondria (panel 1 and 3), whereas the mutant parkin C2898G does not (panel 2 and 4). Overexpression of DNAJB2a, DNAJB6b, or DNAJB8 did not rescue parkin C289G relocation to mitochondria (panel 5-7). Squares indicate the areas that are magnified in the inset to show overlap or no overlap between parkin and the mitochondrial staining. (B) Quantification of the relocation of parkin to mitochondria and the effect of chaperones, normalized to parkin WT treated with CCCP (*=p<0.05; **=p<0.01; ***=p<0.001; n>3 independent samples, mean ± SEM).
